# Population Movement Patterns Among the Democratic Republic of the Congo, Rwanda, and Uganda During an Outbreak of Ebola Virus Disease: Results from Community Engagement in Two Districts — Uganda, March 2019

**DOI:** 10.15585/mmwr.mm6901a3

**Published:** 2020-01-10

**Authors:** Lydia Nakiire, Herman Mwanja, Satish K. Pillai, Jonan Gasanani, Dickson Ntungire, Stephen Nsabiyumva, Richardson Mafigiri, MID, Nick Muneza, Sarah E. Ward, Zeinabou Daffe, Peter Babigumira Ahabwe, Simon Kyazze, MBAIT, Joseph Ojwang, Jaco Homsy, Elvira Mclntyre, Mohammed Lamorde, Richard Walwema, Issa Makumbi, Allan Muruta, Rebecca D. Merrill

**Affiliations:** ^1^Infectious Diseases Institute, Makerere University, Kampala, Uganda; ^2^National Center for Emerging and Zoonotic Infectious Diseases, CDC; ^3^Ministry of Health, Kampala, Uganda; ^4^Center for Global Health, CDC; ^5^Agency for Toxic Substances and Disease Registry, U.S. Department of Health and Human Services.

Tailoring communicable disease preparedness and response strategies to unique population movement patterns between an outbreak area and neighboring countries can help limit the international spread of disease. Global recognition of the value of addressing community connectivity in preparedness and response, through field work and visualizing the identified movement patterns, is reflected in the World Health Organization’s declaration on July 17, 2019, that the 10th Ebola virus disease (Ebola) outbreak in the Democratic Republic of the Congo (DRC) was a Public Health Emergency of International Concern (*1*). In March 2019, the Infectious Diseases Institute (IDI), Uganda, in collaboration with the Ministry of Health (MOH) Uganda and CDC, had previously identified areas at increased risk for Ebola importation by facilitating community engagement with participatory mapping to characterize cross-border population connectivity patterns. Multisectoral participants identified 31 locations and associated movement pathways with high levels of connectivity to the Ebola outbreak areas. They described a major shift in the movement pattern between Goma (DRC) and Kisoro (Uganda), mainly through Rwanda, when Rwanda closed the Cyanika ground crossing with Uganda. This closure led some travelers to use a potentially less secure route within DRC. District and national leadership used these results to bolster preparedness at identified points of entry and health care facilities and prioritized locations at high risk further into Uganda, especially markets and transportation hubs, for enhanced preparedness. Strategies to forecast, identify, and rapidly respond to the international spread of disease require adapting to complex, dynamic, multisectoral cross-border population movement, which can be influenced by border control and public health measures of neighboring countries

During March 15–25, 2019, IDI and CDC, on behalf of MOH Uganda, assessed population movement patterns using the Population Connectivity Across Borders (PopCAB) toolkit,* a CDC innovation, in Uganda’s southwestern Kanungu and Kisoro districts bordering DRC and Rwanda (*2*). Qualitative and spatial data were collected using community-level focus group discussions and key informant interviews with participatory mapping to characterize cross-border population movement patterns, through which participants helped facilitators annotate points of interest and travel routes on printed maps scaled to show the tricountry area (DRC, Rwanda, and Uganda). The team purposively sampled participants and event locations to ensure multisectoral representation and incorporate principal locations along community-level movement patterns based on contextual knowledge and discussions with district- and community-level leaders. The IDI-CDC team analyzed the qualitative data to identify themes addressing cross-border movement and border interventions and created and compiled spatial data for all identified locations and travel routes.

The IDI-CDC team conducted 12 data collection events with 52 participants, including border public health volunteers, health care providers, security officials, transportation officials, community leaders, army officers, and informal traders (Table). Participants described movement patterns across the DRC, Rwanda, and Uganda region associated with residents of DRC who were 1) seeking refugee status in Uganda; 2) conducting trade and other business; 3) seeking health care; or 4) visiting family. Participants identified 26 priority locations of interest, and five specific pathways connecting them, including refugee transit centers (two, including one that was also identified as a school), points of entry (eight), health care facilities (six), markets and entrepreneurial sites (nine), schools (one), and transportation hubs (one) (Figure). Participants also identified health care facilities that receive patients traveling from DRC to access cheaper and higher-quality medical care in Uganda. Although participants consistently described refugees as mostly women and children traveling by foot and traders as mostly adults traveling on motorbikes and trucks, the demographics and modes of travel for seeking health care and visiting family were inconsistent.

**TABLE:** Population Connectivity Across Borders field events in Kanungu and Kisoro districts — Uganda, March 2019

Date	Type (no. of participants)	District	Target group	Event location
Mar 15	Key informant interview (1)	Kisoro	Border screening volunteer	Nteko, unofficial POE
Mar 18	Focus group discussion (8)	Kisoro	Transport personnel (motorcycle taxi drivers)	Bunagana, official POE
Mar 18	Key informant interview (1)	Kisoro	Security personnel	Bunagana, official POE
Mar 19	Key informant interview (1)	Kisoro	Health care worker	Nyakabande refugee transit camp
Mar 19	Focus group discussion (4)	Kisoro	Health care workers	Kisoro Hospital
Mar 20	Key informant interview (1)	Kanungu	Security personnel	Kanungu district health office
Mar 21	Key informant interview (1)	Kanungu	District health personnel	Kanungu district health office
Mar 21	Focus group discussion (10)	Kanungu	Community leaders	Butogota (also called Kyeshero), official POE
Mar 22	Focus group discussion (8)	Kanungu	Traders	Ishasha, official POE
Mar 23	Focus group discussion (8)	Kanungu	Health care workers	Bwindi Community Hospital
Mar 25	Focus group discussion (8)	Kanungu	Military personnel at the border point	Kayonza Tea Factory
Mar 25	Key informant interview (1)	Kanungu	Health care worker	Matanda refugee transit center

**Abbreviation:** POE = point of entry.

**FIGURE:**
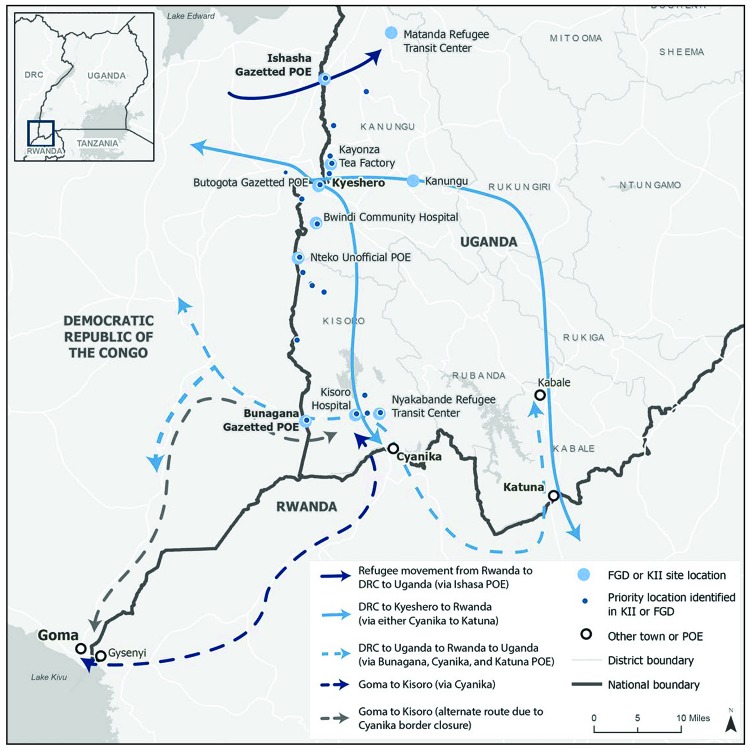
Population movement pathways and points of interest from 12 Population Connectivity Across Borders events — Kanungu and Kisoro districts, Uganda, March 2019 **Abbreviations:** DRC = Democratic Republic of the Congo; FGD = focus group discussion; KII = key informant interview; POE = point of entry.

Five of the 12 events (three focus group discussions and two key informant interviews) described cross-border movements principally between DRC and Uganda, directly and through Rwanda. A main travel pathway linking the three countries was a bus route from DRC (Goma, Butembo, and other areas) through Rwanda (Gisenyi) and then into Uganda via the Cyanika ground crossing to Kisoro district (Figure). This bus route was mainly used to avoid the insecurity within DRC. However, after the border crossings at Cyanika and Katuna were closed indefinitely beginning February 28, 2019, passengers disembarked at the Rwanda-Uganda border, then walked to the Uganda side, where they rode motorcycle taxis (called “boda bodas”) or other buses into Kisoro town (*3*). Some respondents also indicated that bus traffic was increasing on the more direct and insecure route through DRC from Goma, DRC, to Bunagana, Uganda, excluding Rwanda. Another multicountry pathway travelers followed was from DRC through Butogota ground crossing in Kanungu District followed by continued travel to Rwanda using two main routes: 1) southward through the Bwindi forest to Kisoro town and on to Rwanda through Cyanika ground crossing or 2) by bus through Kanungu District, eastward to Rukungiri District, then south to Kabale city, and into Rwanda through Katuna ground crossing. Respondents in the focus group discussions did not describe adjustments to the highlighted southward pathways into Rwanda following the Cyanika and Katuna ground crossing closures, in contrast to the shift in travel patterns from Goma to Uganda.

No seasonality was associated with general population movement, which was almost uniformly described as constant. However, increases in refugee movement were associated with more insecurity in DRC, and increases in trader movements were associated with agricultural cycles for a range of products.

The Uganda MOH National Task Force, which has led Ebola preparedness activities since DRC declared the Ebola outbreak on August 1, 2018, along with district leadership, used these findings to prioritize health facilities, points of entry, and villages for enhanced preparedness activities. These measures included public health screening of travelers, enhanced community-based surveillance procedures, targeted risk communication, and Ebola vaccination of frontline workers at facilities that were more likely to receive patients from outbreak-affected areas of DRC.

## Discussion

This population connectivity mapping exercise in two high-priority districts of southwestern Uganda helped national and district leadership identify locations and population groups to prioritize for preparedness efforts to avert or contain importation of Ebola from DRC. District-level stakeholders identified numerous locations within Uganda with high connectivity to the outbreak area in DRC. Additional locations within Uganda, such as the Kisoro bus park, were highlighted because of their risk for onward transmission to urban areas within southwestern Uganda and more distant locations, including the capital city of Kampala. The results illustrated the impact of border system interventions on population movement patterns, as evidenced by the major shifts in movement following border closures.

Goma, a large urban area in DRC, first reported an Ebola case on July 30, 2019, which led to a cluster with four confirmed cases (*4*). The multisectoral participants described high connectivity between Goma and southwestern Uganda, raising the need to address population movement and connectivity dynamics there. In response, District Health Officers in Kanungu and Kisoro used the PopCAB results to rapidly target preparedness activities and response capacity assessments to highly connected locations.

When adapting preparedness and response initiatives to the range of locations identified through this initiative, national and district leaders considered the unique characteristics of each location. Preparing workers in a marketplace to better identify persons with suspected Ebola cases follows a different process from that of preparing workers in points of entry or health care facilities. Market vendors who typically lack medical or public health training, require sensitization and training suitable to their backgrounds. Of note, the observed markets had multiple, nonuniform entry points, posing challenges to screening all market visitors. To increase the likelihood of identifying persons with possible Ebola at such busy locations or on inbound or outbound routes, preparedness efforts might require sensitizing not only stakeholders at the venues but also communities connected with them by proximity or travel patterns. National border health interventions must evolve to accommodate those implemented in neighboring countries as demonstrated by the highlighted shift in movement between countries caused by the Cyanika ground crossing closure.

The findings in this report are subject to at least one limitation regarding potential biases among the purposive participant sampling during the PopCAB implementation. To reduce the risk for bias, the team invited participants who represented multiple sectors and facilitated events in a range of locations across Kanungu and Kisoro districts.

This multisectoral, community-level engagement in southwestern Uganda helped to characterize the complexity of population movement and connectivity among DRC, Rwanda, and Uganda. National and district leadership in Uganda used the results to guide comprehensive border health strategies including identifying ground crossings for enhanced traveler screening and health facilities for surveillance, both along the border and within Uganda (*2*). In addition, the Uganda National Task Force used the results to tailor its surveillance, infection prevention and control, and risk communication strategies to address geographic areas at risk for importation of Ebola and to incorporate community-level sectors that interact with populations connected to the outbreak areas. This method was also adapted and applied to strengthen preparedness for mass gatherings and Ebola vaccination campaigns. Uganda’s application of the PopCAB method to enhance Ebola preparedness and response initiatives could be adapted by other countries to better integrate multisectoral, cross-border population movement dynamics, especially in response to events in neighboring countries, into a broader response strategy to forecast, identify, and rapidly respond to the international spread of disease.

**Summary**

**What is known about this topic?**

Understanding cross-border population movement patterns can help countries tailor public health interventions to limit international spread of communicable disease.

**What is added by this report?**

Land-based travel routes among the Democratic Republic of the Congo, Rwanda, and Uganda shifted as a result of formal border closures. Uganda assessed population movement patterns to tailor its surveillance, infection prevention and control, and communication strategies to address the risk for importation of Ebola virus disease from neighboring countries.

**What are the implications for public health practice?**

Strategies to forecast, identify, and rapidly respond to the international spread of disease require adapting to complex, dynamic, multisectoral cross-border population movement, which can be influenced by border control and public health measures used by neighboring countries.

## Footnotes

* https://www.cdc.gov/globalhealth/security/ghsareport/2018/cdc-innovation.html.
